# Supplementation of cow milk naturally enriched in polyunsaturated fatty acids and polyphenols to growing rats

**DOI:** 10.1371/journal.pone.0172909

**Published:** 2017-03-07

**Authors:** Nadine W. Santos, Emerson H. Yoshimura, Cecília E. Mareze-Costa, Erica Machado, Bruna C. Agustinho, Lucelia M. Pereira, Márcia N. Brito, Nilton A. Brito, Lucia M. Zeoula

**Affiliations:** 1 Departamento de Zootecnia, Universidade Estadual de Maringá, Maringá, Brazil; 2 Departamento de Ciências Fisiológicas, Universidade Estadual de Maringá, Maringá, Brazil; Laval University, CANADA

## Abstract

This study investigated whether intake of cow milk, naturally enriched with polyunsaturated fatty acids (PUFA, omega-3) and polyphenols (from propolis extract and vitamin E), from manipulation of cow’s diet, would result in positive metabolic effects in rats from weaning until adulthood. Male *Wistar* rats were fed a standard chow diet or a hypercaloric diet (metabolically disturbed rats, obese) which was supplemented with either whole common milk, milk enriched with PUFA (PUFA-M) or milk enriched with PUFA and polyphenols (PUFA/P-M), at 5mL/kg body weight,having water as control. Whole milk supplementation increased initial weight gain and reduced gain in the adulthood of rats. Intake of common milk reduced cholesterol levels in non-obese rats and reduced insulin resistance in obese rats. PUFA-milk showed a decreasing effect on plasma triacylglycerol and VLDL concentrations, increasing plasma HDL concentration and reducing adipocyte size of non-obese rats, but no effect was observed in obese rats. PUFA/P-milk in obese rats resulted in greater deposition of muscle mass and mesenteric fat, with a tendency to lower LDL levels, and resulted a visceral fat accumulation in non-obese rats. Thus, whole common milk and PUFA-rich milk have shown to be beneficial in a normal metabolic condition, whereas common milk and milk enriched with PUFA and polyphenols improve metabolic effects of obesity.

## Introduction

Cow milk is a common and important food in human diet, especially for children and older people. Milk and dairy products are sources of nutrients and provide energy, high quality protein and essential vitamins and minerals [1]. Consumption of cow milk has been criticized by nutritionists as fat content is mainly composed of saturated fatty acids (SFA). Consumption of saturated fatty acids has been linked to occurrence of heart disease [2] and, because of this association, consumption of cow milk has also been identified as a critical contributing dietary factor. The association of milk with adverse effects has not been confirmed [3], however, it has still been highly publicized.

Studies have investigated ways to improve the milk fat profile through animal nutrition. Lipid supplementation of cows, especially with feeds rich in polyunsaturated fatty acids (PUFA) may increase proportion of PUFAs and conjugated linoleic acid (CLA) in milk, with potential benefits for human health [1]. Studies have shown that consumption of omega-3 PUFA-rich foods have hypolipidemic, antithrombotic and anti-inflammatory effects [4] and CLA may enhance insulin sensitivity and reduce adiposity [5]. Thus, intake of PUFA-enriched milk could aid in prevention of metabolic disorders of acquired obesity throughout life.

Unfortunately, high concentrations of PUFA in milk modify the stability of fat, which may then oxidize and develop a rancid taste and odor [6], reducing the time available for consumption compared to milk with a common fat profile. For this reason, efforts have been made to enrich milk with antioxidant compounds, e.g. polyphenols, in a natural way by altering the feeding of cows [7,8]. Antioxidant activity of milk may be increased by providing to cow a diet containing a propolis-based additive, which is rich in plant polyphenols [9], and also a propolis-based additive associated with vitamin E in diets [10].

Milk enrichment is an interesting subject, as milk is a commonly consumed food and has potential to easily contribute a greater amount of beneficial compounds in diet, without altering food patterns. Effects of human consumption of milk enriched with PUFA, produced via supplementation of the diet of cows, has been previously reported [11], however, consequences of consumption of milk enriched with PUFA and polyphenols with antioxidant capacity remains unknown. Also considering that dietary antioxidant transfer into milk is not high [10] and may increase feeding cost, it is important to ascertain whether milk enriched with polyphenols can bring effects to those who consume it. The objective of this study was to determine the effects of supplying milk enriched with PUFA and polyphenols on feed intake, growth, blood parameters, glucose tolerance and body composition of non-obese rats and diet-induced obese rats.

## Materials and methods

### Animals and diets

This study was approved by the Ethics Committee for the Use of Animals in Experiments of the State University of Maringá (Maringá, Paraná, Brazil), statement number 115/2012. Animals were from the colony of central animal house of this institution.

Eighty 21-day-old male Wistar rats (*Rattus norvegicus*) were randomly distributed into cages (46 × 24 × 20 cm) containing five animals per cage, with each animal having an average initial body weight of 78.5g. Rats were maintained at 24°C with a 12-hour light-dark cycle, and were provided *ad libitum* feed and water. Forty rats were fed a standard chow diet while, in parallel, forty rats were fed a hypercaloric diet to induce metabolic disorders of obesity. Standard chow diet was based on Nuvilab CR1 ration (Nuvital^®^, Colombo, Paraná, Brazil), composed by 879.7 g/kg of dry matter (DM), 261.6 g/kg of crude protein, 22.9 g/kg of ether extract and 3,959 kcal/kg of gross energy. Hypercaloric diet was composed by standard ration (514 g/kg), sweetened condensed milk (330 g/kg), sugar (70 g/kg), and water (86 g/kg) according to Lima et al. [12], and presented (g/kg of DM): 760.9 g/kg of DM, 156.5 g/kg of crude protein, 53.1 g/kg of ether extract and 4,303.2 kcal/kg of gross energy.

Milk supplied to rats was obtained from dairy cows used for experimentation at the Experimental Farm of Iguatemi of the State University of Maringá. Cows were fed a basal diet which was supplemented with flaxseed oil (rich in PUFA omega-3, 25 g/kg DM), without or with polyphenols (propolis extract, 1.2 g/kg DM, and vitamin E, 375 IU/kg DM), in order to naturally produce milk enriched with PUFA or PUFA and polyphenols as previously described by Santos et al. [10]. Milk composition is presented in Table 1. After milking the cows, raw milk were distributed into polyethylene tubes (12 mL) and frozen. Immediately prior to providing milk to rats, it was thawed (4°C) and vortexed for 5 minutes.

**Table 1:** Composition of common milk (COM-M), milk enriched with polyunsaturated fatty acids (PUFA-M) and milk enriched with PUFA and polyphenols (PUFA/P-M).

Component	Milk			P
	COM-M	PUFA-M	PUFA/P-M	
Chemical composition (mg/mL)				
Fat	29.80 ± 0.1	26.86 ± 0.8	27.78 ± 0.4	0.18
Protein	28.99 ± 0.2	30.74 ± 0.9	29.26 ± 0.6	0.68
Lactose	46.31 ± 0.1	45.81 ± 0.6	47.50 ± 0.2	0.34
Total solids	114.40 ± 0.4	112.59 ± 0.7	113.95 ± 0.8	0.72
Fatty acid composition (mg/mL)				
12:0	0.82 ± 0.04	0.64 ± 0.14	0.67 ± 0.02	0.87
14:0	2.70 ± 0.08	2.53 ± 0.31	2.49 ± 0.01	0.97
16:0	7.02 ± 0.63	5.96 ± 0.01	5.34 ± 0.12	0.52
18:0	1.73 ± 0.48	3.34 ± 0.19	3.28 ± 0.02	0.11
*trans-*9-18:1	0.18 ± 0.10b	0.58 ± 0.09ab	0.70 ± 0.02b	0.03
18:1 (n-9)	4.01 ± 0.77	6.52 ± 0.55	5.89 ± 0.21	0.10
18:2 (n-6)	0.47 ± 0.16	0.66 ± 0.05	0.70 ± 0.01	0.15
18:3 (n-3)	0.06 ± 0.02b	0.30 ± 0.02a	0.29 ± 0.01a	0.004
*cis-*9,*trans-*11-18:2	0.09 ± 0.03	0.12 ± 0.01	0.12 ± 0.01	0.12
*trans-*10,*cis-*12-18:2	0.02 ± 0.01	0.03 ±0.01	0.03 ±0.01	0.35
Total CLA	0.11 ± 0.03	0.15 ± 0.01	0.15 ± 0.01	0.14
SFA	13.98 ± 4.34	14.00 ± 0.43	13.42 ± 0.65	0.98
MUFA	5.43 ± 1.17	8.23 ± 0.52	7.61 ± 0.17	0.14
PUFA	0.67 ± 0.22	1.14 ± 0.09	1.17 ± 0.02	0.04
n-6/n-3	8.61a	2.28b	2.55b	<0.001
Antioxidant quality and oxidative stability				
Total polyphenols (GAE mg/L)^1^	11.33 ± 0.61b	10.16 ± 0.69b	18.15 ± 1.30a	0.03
Orac (TE mmol/L)^2^	10.57 ± 0.29	11.18 ± 0.70	14.70 ± 0.06	0.56
Reducing power (GAE mg/L)	25.85 ± 0.32	35.78 ± 0.57	38.32 ± 0.59	0.05
Conjugated diene (mmol/kg fat)	41.51 ± 0.09b	59.55 ± 0.28a	54.53 ± 0.53ab	0.03
Tbars (MDAE mmol/kg fat)^3^	2.68 ± 0.34	5.28 ± 0.76	3.43 ± 0.89	0.52

Data are mean ± standard deviation. CLA = conjugated linoleic acid, SFA = saturated fatty acids, MUFA = monounsaturated fatty acids. Means with different letters differ by Tukey's test.

^1^GAE = gallic acid equivalent,

^2^TE = Trolox^®^ equivalent,

^3^MDAE = malondialdehyde equivalent.

Eight experimental groups (*n =* 10), four groups of animals fed standard chow diet and four groups fed hypercaloric diet were established. For each type of diet there was a group without supplementation (control with water), a group supplemented with whole common milk (COM-M), a group supplemented with milk enriched with PUFA (PUFA-M) and another group supplemented with milk enriched with PUFA and polyphenols (PUFA/P-M). Milk supplementation was performed daily by gavage at a dose of 5mL/kg of body weight, which was adjusted weekly depending on weight gain of animals. Milk dose provided to rats was calculated based on recommendation by the Brazilian Ministry of Health [13] of 150 liters per year of milk and dairy products for an average weight Brazilian man [14].

### Experimental procedures

Experimental period was a total of 85 days, in a completely randomized design with eight treatments and ten replicates. Experiment began immediately after weaning rats, without a period of adaptation to the diet. Rats were supplemented with milk from weaning (21 days) to avoid risks of lactose intolerance, continuing until 106–110 days, during growth phase and part of adult phase. Offered feed and refusals were weighed daily to determine feed intake. Rats were weighed weekly.

For the intravenous glucose tolerance test, animals were anesthetized (thiopental, 40 mg/kg body weight; i.p.) and subjected to a minor surgery to implant a silicon catheter into the jugular vein. Next day, fasting blood samples (0.1 mL) were collected before glucose infusion (1 g/kg), and at 5, 15, 30 and 60 minutes after infusion. Blood samples were centrifuged (2,500 *g*, 20 minutes, 4°C) and plasma was stored at -12°C for determination of glucose concentrations. Glucose tolerance was evaluated by the area under the curve for obtained glucose values.

Intraperitoneal insulin tolerance test (1 U/kg insulin) was performed on obese animals, blood samples were collected at time 0 (baseline), 15, 30, 60, 120, 240, and 300 minutes after insulin injection. Glucose disappearance rate was calculated using the formula 0.693/t_1/2_. The plasma glucose t_1/2_ was calculated from the slope of least squares analysis of plasma glucose levels during the linear phase of decline curve [15].

At the end of experimental period, animals were anesthetized with sodium thionembutal (thiopental, 40 mg/kg body weight) after an overnight fast, naso-anal length was measured and blood was collected from the inferior vena cava. To obtain the serum, blood was centrifuged (2,500 *g*, 20 minutes, 4°C) and stored (-12°C) for further analysis. Animals were subjected to a laparotomy and, after sacrifice of animals, organs were removed and weighed, including liver, soleus and gastrocnemius muscles and fat deposits (periepididymal, retroperitoneal, mesenteric, subcutaneous and brown fats). Lee index was calculated from the ratio between the cube root of body weight and naso-anal length of rats [16].

### Chemical analysis

The N, fat and lactose concentrations in milk were determined by infrared spectroscopy (Bentley model 2000, Chaska, MN, USA) following procedure 972.16 of AOAC [17]. Concentrations of milk urea N (MUN) were determined by a colorimetric method with the Berthelot reaction (Chemspec 150, Chaska, MN, USA). Milk somatic cell counts (SCC) were obtained using an electronic counter (Somacount 500^®^, Chaska, MN, USA)

Total lipids in milk were extracted according to Folch et al. [18]. Methyl esters of fatty acids were prepared following the Hartman and Lago procedure [19]. Methyl esters of fatty acids were separated and quantified using gas chromatography (Thermo, Trace GC Ultra 3300, Waltham, MA, USA) according to Simionato et al. [20]. Results were expressed as mg/mL of milk. DM content of feed was determined using a forced air oven, according to the AOAC procedure 934.01 [17].

Polyphenol extraction was performed from one mL of milk which volume was made up to 10 mL with pure methanol, protected from light, and vortex-mixed for one minute. This extract was centrifuged (1058g, 10 min, 20°C) and filtered on a 0.22 mm polytetrafluoroethylene membrane filter (Spritzen, Shanghai, China) as described by Santos et al. [8]. Total polyphenol content was determined using the Folin-Ciocalteu procedure [21] by mixing 125 μL of sample with 125 μL of Folin-Ciocalteu solution (500:500, v/v) and 2.250 μL of Na_2_CO_3_ (3.79 M). After incubation at room temperature (*i*.*e*., 23°C) for 30 min, reads were performed in a spectrophotometer (PC 300 Thermo Scientific, Waltham, MA, USA), and results were expressed as gallic acid equivalent (GAE; mg/L). Total antioxidant capacity of milk samples was determined using the oxygen radical absorbance capacity (ORAC) method according to Zulueta et al. [22] and results were expressed as mmol of Trolox equivalent (TE/L). Total reducing power was determined [23] with some modifications [8] and results were reported as GAE (mg/L). Lipid oxidation in milk was evaluated by measuring the concentration of conjugated diene hydroperoxides (CD; mmol/kg fat) [24], and thiobarbituric acid reactive substances (TBARS) as described by Vyncke [25], expressed as malondialdehyde-equivalents (MDAE/kg fat).

Blood concentrations of glucose, total cholesterol, HDL cholesterol, triacylglycerol, aspartate aminotransferase and alanine aminotransferase were determined using colorimetric methods (Gold Analisa^®^, Belo Horizonte, MG, Brazil) and measured on a spectrophotometer (Bioplus2000^®^, São Paulo, SP, Brazil). LDL cholesterol level was estimated by the Friedewald equation; LDL cholesterol (mg/100mL) = total cholesterol—HDL—(triacylglycerol/2.2). Oxidation of blood proteins was assessed by determination of reduced thiols [26]. The ABTS radical (2,2'-azino-bis[3-ethylbenzothiazoline-6-sulphonic acid]) was used to analyze the total antioxidant capacity of blood [27]. Adipocytes were isolated and the diameter was determined according to Rodbell [28], with modifications by Foletto et al. [29].

### Statistical analysis

Results were analyzed using the generalized linear model procedure of SAS 9.0 (SAS Institute, Cary, NC, USA) with a variance analysis, and significance declared at *P*<0.05, and a tendency to be significant was accepted at *P<*0.10. Differences in chemical composition between the three type of milk were compared using Tukey’s test. For all other variables measured, orthogonal contrasts were used to compare effects between groups: 1) milk supplementation (control vs. COM-M, PUFA-M and PUFA/P-M); 2) supplementation with PUFA-milk (COM-M vs. PUFA-M and PUFA/P-M); and 3) supplementation with PUFA/P-milk (PUFA-M vs. PUFA/P-M). Student's t-test was used to compare means from control groups of non-obese and obese rats.

## Results

Milk supplementation (common, enriched with PUFA and enriched with PUFA and polyphenols) in diets of non-obese and obese rats resulted in significant differences and tendencies in several metabolic parameters evaluated. However, some data were suppressed because there was no effect of milk supplementation on obese disturbed rats.

Animals fed hypercaloric diet had significantly higher final body weight than animals fed standard chow diet (Table 2), with 8% increase of weight in control group (*P* = 0.0083), 7% in COM-M (*P* = 0.0383), 10% in PUFA-M (*P* = 0.0329) and 11% in PUFA/P-M (*P* = 0.01). Common milk supplementation did not affect feed intake, final weight, naso-anal length or Lee index of non-obese rats compared to control (water). Obese rats supplied with milk had lower feed intake (*P* = 0.01) and tended to have minor naso-anal length, compared to control. PUFA/A-M tended to increase the Lee index (*P* = 0.06) of obese rats.

**Table 2:** Feed intake, body weight, length and Lee index of non-obese rats and obese rats fed a diet supplemented with common milk (COM-M), milk enriched with polyunsaturated fatty acids (PUFA-M) or milk enriched with PUFA and polyphenols (PUFA/P-M).

Parameters	Non-obese rats rats				P^1^		
	Control	COM-M	PUFA-M	PUFA/P-M	1	2	3
Feed intake (g/day)	36.10 ± 2.95	33.10 ± 2.33	34.60 ± 2.27	38.00 ± 11.65	0.79	0.36	0.40
Initial body weight (g)	78.40 ± 9.91	77.30 ± 8.65	78.95 ± 8.72	79.35 ± 10.93	0.97	0.62	0.93
Final body weight (g)	376.65 ± 18.60	364.85 ± 28.58	358.10 ± 34.20	357.45 ± 35.96	0.14	0.55	0.96
Naso-anal length (cm)	23.70 ± 1.01	23.28 ± 0.83	23.00 ± 0.89	23.07 ± 0.75	0.12	0.47	0.86
Lee index ^2^	304.98 ± 9.13	306.87 ± 5.36	308.68 ± 9.28	307.37 ± 6.16	0.35	0.70	0.70
Parameters	Obese rats				P^1^		
	Control	COM-M	PUFA-M	PUFA/P-M	1	2	3
Feed intake (g/day)	42.40 ± 4.48	32.60 ± 9.69	32.70 ± 3.38	35.70 ± 4.28	0.01	0.63	0.44
Initial body weight (g)	75.90 ± 9.78	78.05 ± 9.97	76.85 ± 9.20	77.56 ± 13.30	0.68	0.84	0.89
Final body weight (g)	406.90 ± 26.39	391.25 ± 27.20	393.25 ± 33.84	397.44 ± 25.31	0.23	0.72	0.76
Naso-anal length (cm)	23.79 ± 0.71	23.43 ± 0.69	23.51 ± 0.46	23.20 ± 0.66	0.09	0.61	0.18
Lee index ^2^	311.53 ± 8.98	312.09 ± 4.58	311.38 ± 5.36	316.84 ± 3.29	0.38	0.37	0.06

Data are mean ± standard deviation.

^1^Probability of significant orthogonal contrasts. Effects tested using orthogonal contrasts were between: 1) Control vs. COM-M, PUFA-M and PUFA/P-M; 2) COM-M vs. PUFA-M and PUFA/P-M; and 3) PUFA-M vs. PUFA/P-M.

^2^Ratio between cubic root of body weight and naso-anal length.

**Fig 1:**
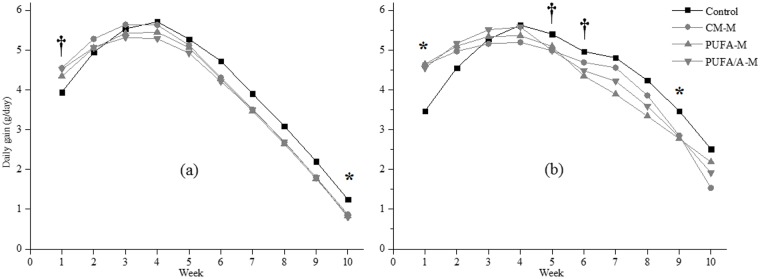
Daily weight gain of (a) non-obese rats and (b) obese rats supplemented with common milk (CM-M), milk enriched with polyunsaturated fatty acids (PUFA-M) and milk enriched with PUFA and polyphenols (PUFA/A-M). * *P < 0*.*05*, †*P < 0*.*10*, *control vs*. *CM-M*.

Non-obese and obese rats had higher body weight gain in the first four weeks after weaning, a period of high growth (Fig 1). Then, there was a more pronounced decrease in non-obese rats compared to obese rats. Non-obese rats supplied with milk tended to gain more weight (*P* = 0.09) at week one, and showed a significant decrease (*P* = 0.03) in weight gain at week 10. Similarly, in obese rats milk increased weight gain at week one (*P* = 0.001) and tended to reduce gain at week five (*P* = 0.06) and six (*P* = 0.06), with a significant decrease at week nine (*P* = 0.01).

In non-obese rats, supplementation with milk caused a decrease in plasma levels of total cholesterol (*P* = 0.004) and LDL cholesterol (*P* = 0.001) (Table 3). Non-obese rats that received supplementation with PUFA-milk, compared to those receiving common milk, had lower triacylglycerol (*P* = 0.005) and VLDL (*P* = 0.005) levels, and higher HDL (*P* = 0.01) and LDL (*P* = 0.005) cholesterol. Giving PUFA/P-milk, compared to PUFA-milk, tended to increase total cholesterol (*P* = 0.08) and LDL cholesterol (*P* = 0.06) levels. Values for blood glucose, aspartate transaminase, alanine aminotransferase, reduced thiols and total antioxidant capacity were unchanged in rats fed diets supplemented with milk. In obese rats, common or enriched milk did not changed blood parameters, except for LDL-cholesterol which was decreased by PUFA/A-milk (*P* = 0.08) (data not shown).

**Table 3:** Plasma biochemical profile of non-obese rats fed a diet supplemented with common milk (COM-M), milk enriched with polyunsaturated fatty acids (PUFA-M) or milk enriched with PUFA and polyphenols (PUFA/P-M).

Parameters/	Non-obese rats				P^1^		
	Control	COM-M	PUFA-M	PUFA/P-M	1	2	3
Glucose (mg/100 mL)	84.30 ± 6.07	88.10 ± 10.21	87.50 ± 9.98	88.70 ± 5.91	0.22	1.00	0.75
Triacylglycerol (mg/100 mL)	83.51 ± 19.34	109.18 ± 21.97	77.06± 15.73	82.71 ± 23.54	0.47	0.005	0.59
Total cholesterol (mg/100 mL)	93.69 ± 11.52	74.83 ± 12.54	78.06 ± 10.46	87.81 ± 14.16	0.004	0.10	0.08
VLDL (mg/100 mL)	16.70 ± 3.87	21.84 ± 4.39	15.41 ± 3.15	16.54 ± 4.71	0.47	0.005	0.59
HDL (mg/100 mL)	36.74 ± 3.93	31.91 ± 7.19	37.39 ± 5.82	41.61 ± 7.95	0.93	0.01	0.20
LDL (mg/100 mL)	40.25 ± 5.04	21.08 ± 2.25	25.26 ± 3.78	29.66 ± 3.13	0.001	0.005	0.06
AST (mg/100 mL)^2^	96.61 ± 13.37	97.72 ± 16.62	91.23 ± 5.92	93.07 ± 20.51	0.74	0.47	0.83
ALT (mg/100 mL)	30.78 ± 5.46	27.49 ± 7.08	31.20 ± 6.57	28.25 ± 6.40	0.55	0.46	0.40
Reduced thiols (nmol/mg protein)	177.94 ± 22.93	180.33 ± 18.03	189.45 ± 28.34	197.64 ± 19.13	0.35	0.30	0.57
TAC (μmol/mg protein)	3.02 ± 0.59	3.08 ± 0.42	3.17 ± 0.38	3.01 ± 0.15	0.72	0.96	0.56

Data are mean ± standard deviation. AST = aspartate transaminase, ALT = alanine aminotransferase, TAC = total antioxidant capacity.

^1^Probability of significant orthogonal contrasts. Effects tested using orthogonal contrasts were between: 1) Control vs. COM-M, PUFA-M and PUFA/P-M; 2) COM-M vs. PUFA-M and PUFA/P-M; and 3) PUFA-M vs. PUFA/P-M.

Blood glucose values obtained during glucose tolerance test in non-obese rats are shown in Table 4. There was no difference in blood glucose between control rats and rats supplemented with common milk. PUFA/P-milk increased blood glucose at 15 minutes (*P* = 0.05), and also tended to be increased at 60 minutes (*P* = 0.09) and area under the curve (*P* = 0.08), compared to PUFA-milk. Glucose tolerance test of obese rats was not affected by milk supplementation (data not shown).

**Table 4:** Blood concentrations of glucose during glucose tolerance test in non-obese rats fed a diet supplemented with common milk (COM-M), milk enriched with polyunsaturated fatty acids (PUFA-M) or milk enriched with PUFA and polyphenols (PUFA/P-M).

Glucose (mg/100 mL)	Non-obese rats				P^1^		
	Control	COM-M	PUFA-M	PUFA/P-M	1	2	3
0 minute	96.80 ± 10.38	91.43 ± 6.82	91.75 ± 10.39	94.79 ± 7.35	0.30	0.66	0.53
5 minutes	303.71 ± 24.17	297.17 ± 28.69	308.26 ± 25.84	314.06 ± 31.82	0.82	0.29	0.70
15 minutes	137.54 ± 24.33	130.27 ± 19.58	127.11 ± 23.63	150.14 ± 14.66	0.85	0.40	0.05
30 minutes	78.23 ± 6.75	73.31 ± 11.22	69.65 ± 11.78	76.50 ± 12.10	0.29	0.96	0.24
60 minutes	92.02 ± 8.70	90.11 ± 6.97	86.63 ± 10.81	95.83 ± 12.16	0.79	0.81	0.09
Mean	141.66 ± 10.60	136.46 ± 11.12	136.67 ± 14.16	146.26 ± 9.81	0.71	0.36	0.13
Area under the curve	7376.33 ± 579.07	7173.00 ± 618.79	7047.83 ± 864.40	7657.33 ± 657.62	0.62	0.46	0.08

Data are mean ± standard deviation.

^1^Probability of significant orthogonal contrasts. Effects tested using orthogonal contrasts were between: 1) Control vs. COM-M, PUFA-M and PUFA/P-M; 2) COM-M vs. PUFA-M and PUFA/P-M; and 3) PUFA-M vs. PUFA/P-M.

**Fig 2:**
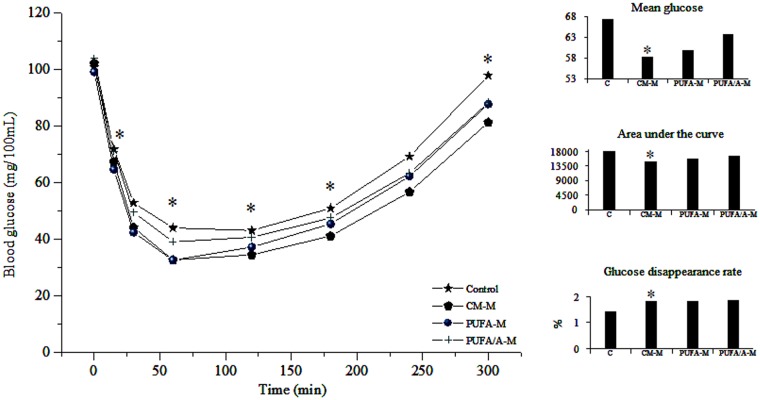
Glucose values in insulin tolerance test of obese rats supplemented with common milk (CM-M), milk enriched with polyunsaturated fatty acids (PUFA-M) and milk enriched with PUFA and polyphenols (PUFA/A-M). *P < 0.*05 control vs*. *CM-M*.

Dietary supplementation of obese rats with milk reduced insulin resistance (Fig 2), there was a significant reduction in glucose values after administration of insulin at times 15 (*P* = 0.001), 60 (*P* = 0.001), 120 (*P* = 0.07), 180 (*P* = 0.048) and 300 minutes (*P* = 0.002), and mean glucose value (*P* = 0.002), also area under the curve (P = 0.002). Glucose disappearance rate increased significantly with milk supplementation (P = 0.002). Enriched milk did not influence this parameter.

Control rats fed hypercaloric diet showed no difference in liver weight, muscle (gastrocnemius and soleus) and brown adipose tissue, compared to rats receiving standard diet (Table 5). However, there was a 46% increase in periepididimal (*P* = 0.0006) and retroperitoneal fat (*P* = 0.0004), 36% in subcutaneous fat (*P* = 0.01), 68% in mesenteric fat and 11% in adipocyte diameter (*P* = 0.0001). Similar results were observed in the other groups when comparing standard diet versus high-sugar diet, confirming diet-induced obesity. Common milk supplementation did not change organ weight relative to body weight. In non-obese rats, PUFA/P-milk increased the weight of periepididymal (*P* = 0.003), retroperitoneal (*P* = 0.017) and subcutaneous (*P* = 0.031) fats relative to body weight when compared to PUFA-milk. PUFA-milk reduced adipocyte diameter (*P* = 0.03) compared to common milk, whereas PUFA/P-milk tended to increase (*P* = 0.08) adipocyte diameter compared to PUFA-milk. In obese rats, rats supplied with common milk tended to present lower adipocyte diameter (*P* = 0.07) than control. PUFA-milk provided larger mesenteric fat (*P* = 0.05) of obese rats. PUFA/A-milk resulted higher weight of gastrocnemius (*P* = 0.03) and a tendency for soleus muscle (*P* = 0.08), besides the tendency to increase retroperitoneal fat (*P* = 0.08) in obese rats.

**Table 5:** Organ and fatty tissue weights relative to body weight of non-obese and obese rats fed a diet supplemented with common milk (COM-M), milk enriched with polyunsaturated fatty acids (PUFA-M) or milk enriched with PUFA and polyphenols (PUFA/P-M).

Parameters	Non-obese rats				P^1^		
	Control	COM-M	PUFA-M	PUFA/P-M	1	2	3
Liver (g/100g)	3.68 ± 0.23	3.47 ± 0.27	3.55 ± 0.27	3.68 ± 0.23	0.23	0.36	0.65
Gastrocnemius muscle (g/100g)	0.58 ± 0.12	0.53 ± 0.15	0.58 ± 0.11	0.58 ± 0.12	0.35	0.65	0.21
Soleus muscle (g/100g)	0.04 ± 0.01	0.05 ± 0.01	0.04 ± 0.01	0.04 ± 0.01	0.65	0.13	0.64
Periepididymal fat (g/100g)	1.12 ± 0.20	1.17 ± 0.21	1.09 ± 0.14	1.12 ± 0.20	0.23	0.34	0.003
Retroperitoneal fat (g/100g)	1.33 ± 0.29	1.38 ± 0.28	1.25 ± 0.33	1.33 ± 0.29	0.51	0.69	0.017
Subcutaneous fat (g/100g)	1.10 ± 0.28	1.08 ± 0.17	0.99 ± 0.26	1.10 ± 0.28	0.99	0.76	0.031
Brown fat (g/100g)	0.066 ± 0.016	0.067 ± 0.011	0.068 ± 0.017	0.066 ± 0.016	0.68	0.74	0.80
Mesenteric fat (g/100g)	0.53 ± 0.12	0.53 ± 0.12	0.52 ± 0.15	0.53 ± 0.12	0.81	0.70	0.41
Adipocyte diameter (μm)	40.68 ± 1.66	43.36 ± 5.51	37.50 ± 1.61	40.68 ± 1.66	0.99	0.03	0.08
Parameters	Obese rats				P^1^		
	Control	COM-M	PUFA-M	PUFA/P-M	1	2	3
Liver (g/100g)	3.60 ± 0.34	3.47 ± 0.27	3.63 ± 0.23	3.61 ± 0.23	0.88	0.16	0.95
Gastrocnemius muscle (g/100g)	0.57 ± 0.06	0.55 ± 0.09	0.54 ± 0.11	0.62 ± 0.12	0.79	0.23	0.03
Soleus muscle (g/100g)	0.05 ± 0.01	0.05 ± 0.01	0.04 ± 0.01	0.05 ± 0.01	0.79	0.70	0.08
Periepididymal fat	1.64 ± 0.34	1.53 ± 0.22	1.56 ± 0.22	1.77 ± 0.37	0.93	0.38	0.35
Retroperitoneal fat	1.94 ± 0.30	1.94 ± 0.39	1.86 ± 0.21	2.20 ± 0.31	0.82	0.70	0.08
Subcutaneous fat	1.50 ± 0.38	1.42 ± 0.36	1.28 ± 0.34	1.61 ± 0.38	0.56	0.98	0.13
Brown fat (g/100g)	0.39 ± 0.08	0.41 ± 0.12	0.41 ± 0.11	0.40 ± 0.11	0.68	0.89	0.71
Mesenteric fat	0.89 ± 0.24	0.66 ± 0.16	0.92 ± 0.16	0.82 ± 0.24	0.16	0.05	0.41
Adipocyte diameter (μm)	45.07 ± 2.06	43.88 ± 4.66	45.01 ± 3.42	44.36 ± 6.00	0.07	0.97	0.95

Data are mean ± standard deviation.

^1^Probability of significant orthogonal contrasts. Effects tested using orthogonal contrasts were between: 1) Control vs. COM-M, PUFA-M and PUFA/P-M; 2) COM-M vs. PUFA-M and PUFA/P-M; and 3) PUFA-M vs. PUFA/P-M.

## Discussion

PUFA-milk presented higher content of fatty acid 18:3 (n-3) than common milk, reducing n-6/n-3 ratio as described earlier [30] (Table 2). Higher concentration of total polyphenols observed in milk PUFA/A-M probably decreased the concentration of conjugated dienes (compared to PUFA-milk), thus lowering lipid oxidation, since conjugated dienes are hydroperoxides formed in the primary stages of oxidation [24]. This is the first study report on milk enriched with PUFA and polyphenols, obtained by feeding the cows, as a supplement to non-obese and obese rats since weaning until adulthood. Supplementation of rats with common milk and milk enriched with PUFA resulted in some effects already reported in literature and other effects are discussed below.

Despite there being no change in body weight throughout the entire experimental period (21–106 days), common milk intake caused an increase in weight gain in the first week after weaning, and a reduction in weight gain during adulthood in both animal models—non-obese and obese (Fig 1). This decrease was not attributed to any changes in intestinal absorption (data not shown). Supplying common milk to obese rats reduced feed intake probably due to an inductor effect on satiety. Although saturated fat in milk is associated with risk factors, in this study non-obese rats supplemented with milk had lower total cholesterol (14%) and LDL (37%) compared to control rats (without milk), and same effect has previously been reported with rats, presenting orotic acid as a contributor to the cholesterol lowering effect (30). Another benefit related from milk ingestion is the content of CLA, calcium and vitamin D (31).

Obesity and insulin resistance are factors that predispose to glucose tolerance and increase risks for development of type 2 diabetes mellitus. Supplementation of obese rats with milk improved insulin resistance (Fig 2), being of fundamental importance to avoid the onset of type 2 diabetes mellitus itself and associated diseases. Similarly in a study of obese adults, consumption of dairy products for 12 weeks improved insulin resistance associated with reduction of oxidative stress [31].

The observed 27% reduction in plasma triacylglycerol and VLDL levels, and increase in plasma HDL and LDL cholesterol in non-obese rats supplemented with PUFA-milk (Table 3), compared to common milk, may be associated with the enriched fat with n-3 fatty acids in this milk (Table 1). This result is similar to those on plasma triacylglycerol and VLDL concentrations of humans supplemented with fish oil rich in n-3 fatty acids [32,33]. Similar observation was earlier attributed to a transition of LDL to larger particles, probably less-atherogenic [34]. Supplementation with PUFA-milk in this group was found to reduce adipocyte size (Table 5). This result is consistent with former findings of a CLA product (*cis-*9, *trans-*11-18:2 and *trans-*10,*cis-*12-18:2) provided to rats [35]. Cows can secrete many isomers of CLA in milk (32) and such food is one of the main sources of naturally synthesized CLA.

PUFA/A-M promoted an increased blood circulation of lipids in non-obese rats, due to the increase in LDL (17%, *P* = 0.06) and HDL levels (11%), although the latter was not significant (*P* = 0.20) (Table 3). Elevation on this lipoproteins, with no effect on VLDL, is consistent with reports of dietary intervention on particle size of LDL, which an increased particle size may be accompanied by elevation of LDL and HDL levels [34]. Polyphenols in milk may have a similar action to Oolong tea, rich in gallic acid and catechins, whose intake significantly reduced particle size of LDL in humans [36]. Thus, such lipoproteins would be of low atherogenicity.

Consumption of PUFA/P-milk increased deposition in periepididymal, retroperitoneal and subcutaneous fat depots in non-obese rats by 28% compared to PUFA-milk (Table 5). Considering that PUFA/P-milk differed from PUFA-milk (Table 1) in total polyphenols, they may be the likely cause for the observed results. Polyphenols present in milk are metabolites mainly from dietary polyphenols metabolized in cow's rumen [37]. In this study, cows received an extract of green propolis, which contained, for example, the Artepillin-C [10], 3,5-diprenyl-4-hidroxycinnamic acid, which was probably transferred into milk as metabolites. Polyphenols can act as activators of cell differentiation of adipocytes as reported by Choi et al. [38], in which treatment of 3T3-L1 cells with Artepillin-C activated the transcription factor of peroxisome proliferator-activated (PPARγ), a key factor in adipogenesis, stimulating cell differentiation and improving glucose transport in adipocytes. Artepillin-C was also able to block the inhibitory effects of tumor necrosis factor (TNF-α), a cytokine involved in adipocyte dysfunction [38].

In study by Ikeda et al. (37), compounds extracted from Brazilian propolis also showed a TNF-α inhibition in 3T3-L1 cells; Artepillin-C was the main compound able to increase PPARγ transcription activity by modulating gene expression of adiponectin. Such observed effects were attributed to prenyl groups at positions three and five in the molecule of cinnamic acid derivatives [39]. Artepillin-C metabolites present in milk may have presented a similar stimulating effect of adipogenesis in adipose tissue, but would not generate inflammatory responses in the body. Therefore such effect of milk enriched with PUFA and polyphenols on inflammatory compounds needs to be investigated in further studies.

Milk enriched with polyphenols showed a hypolipidemic effect and increased muscle mass in obese rats (Table 5). In a similar study with obese rats, supplementation with phenolic compounds (flavonoids, phenolic acids and phytosterols) from fruit extract caused a similar result with lower LDL levels [40]. This effect may be related to the antioxidant action of phenolic compounds (39) in reducing inflammatory reactions characteristic of obesity and hyperlipidemia. Changes in body composition with increased fat mass and decreased lean muscle mass are commonly observed in aging [41]. This condition associated with subclinical inflammation induced by obesity may contribute to development of sarcopenia [42]. Thus, increase in lean muscle mass, observed in the present study, with supplementation of milk enriched with omega-3 and antioxidants may reduce deterioration and muscular dysfunction observed in obesity.

## Conclusion

Providing whole common milk to non-obese and obese rats was beneficial in reducing weight gain in adulthood for both, yet reduced cholesterol levels in non-obese rats and decreased insulin resistance in obese rats.

Milk enriched with PUFA resulted in a positive decrease in VLDL and triglycerides, increase in HDL, with reduction of adipocyte diameter in non-obese rats.

Milk enriched with PUFA and polyphenols increased lean mass of obese rats with a tendency to decrease LDL levels.

Whole common milk shown to be more beneficial in a normal metabolic condition and metabolic disorders of obesity can be attenuated by both intake of whole milk or milk enriched with PUFA and polyphenols.
